# Supplementary material for: The Ycf48 accessory factor occupies the site of the oxygen-evolving manganese cluster during photosystem II biogenesis
Source: Nat Commun. 2023 Aug 4;14:4681. doi: 10.1038/s41467-023-40388-6 (PMC10403576; doi:10.1038/s41467-023-40388-6)
Supplement: Supplementary file 1 — Supplementary Information [file 41467_2023_40388_MOESM1_ESM.pdf]

Supplementary information for

The Ycf48 accessory factor occupies the site of the oxygen-evolving manganese cluster during photosystem II biogenesis

Zhao et al

consisting of:

Supplementary Figures 1-13
Supplementary Tables 1-3
Uncropped Supplementary Fig.2 and Fig.3

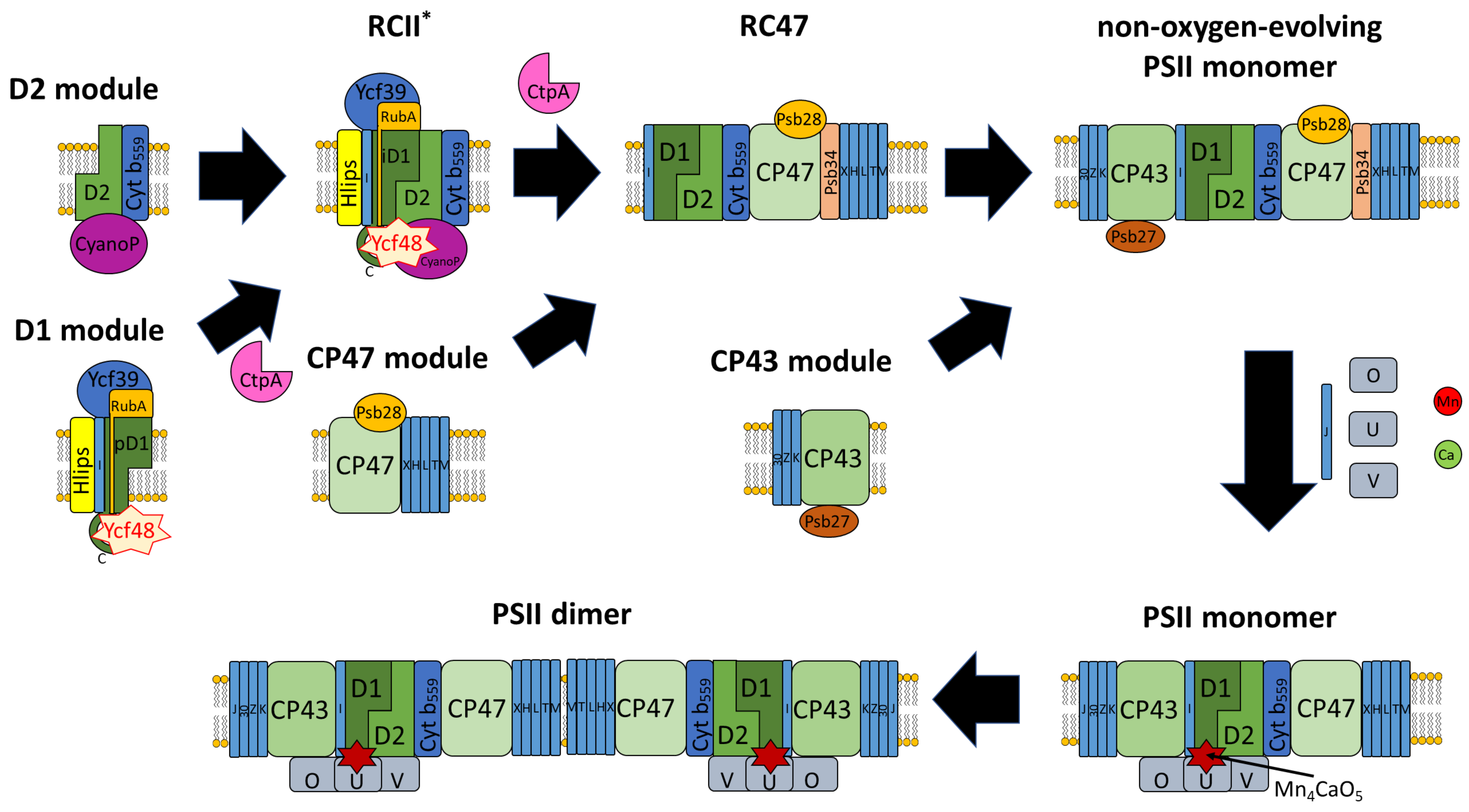

Supplementary Fig. 1 Model for assembly of PSII in cyanobacteria. The D1 module (consisting of precursor D1 (pD1), PsbI, Ycf48, RubA and attached Ycf39/Hlips complex) combines with the D2 module (consisting of D2, cytochrome *b*-559 (Cyt b_{559}) and CyanoP) to form the PSII reaction center assembly complex, RCII*. Subsequent addition of the CP47 module to form the RC47 complex is followed by addition of the CP43 module to form the non-oxygen-evolving PSII monomer, which can then assemble the Mn_4CaO_5 oxygen-evolving cluster and bind PsbJ and the PsbO, PsbU and PsbV extrinsic proteins to form the oxygen-evolving monomeric PSII complex which can then dimerise. PSII subunits are shown without the Psb designation. CtpA is the D1 C-terminal processing protease. Ycf48, CyanoP, RubA, Psb27, Psb28, Psb34 are accessory proteins involved at specific stages of assembly but absent from the final active PSII complex. Whether Psb34 is also a component of the CP47 module is unresolved.

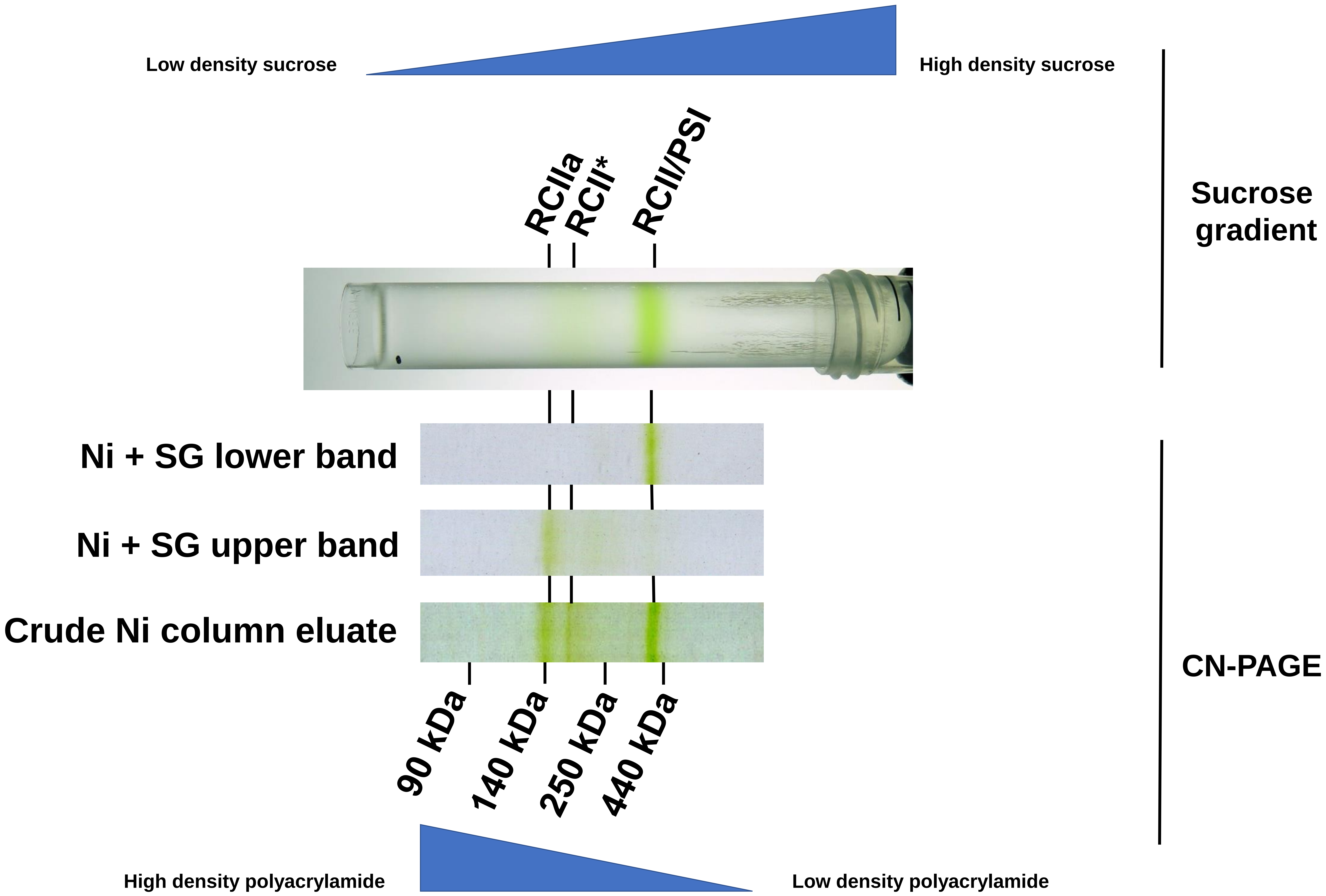

Supplementary Fig. 2 Isolation of the RCII/PSI complex. His-tagged D2 complexes isolated by Ni-affinity chromatography from a strain of Syn6803 lacking CP47 (Ni) were subjected to sucrose-density-gradient centrifugation (Sucrose gradient). The lower band in the sucrose gradient (Ni+SG lower band) contains the RCII/PSI complexes analyzed by cryo-EM while the upper band (Ni+SG upper band) is a mixture of RCIIa and RCII*. The purity of the samples was assessed by clear-native PAGE (CN-PAGE) in the presence of n-dodecyl-β-D-maltoside (DM) and sodium deoxycholate (see methods). Approximately 1 µg of Chl of the crude Ni column preparation was loaded onto the sucrose gradient and the CN gel and 0.5 µg of Chl was loaded onto the CN gel for both the lower sucrose gradient band (Ni + SG lower band) containing the RCII/PSI complex and the upper sucrose gradient band (Ni + SG upper band). This purification was repeated at least four times independently with the same results. Source data are provided at the end of this file.

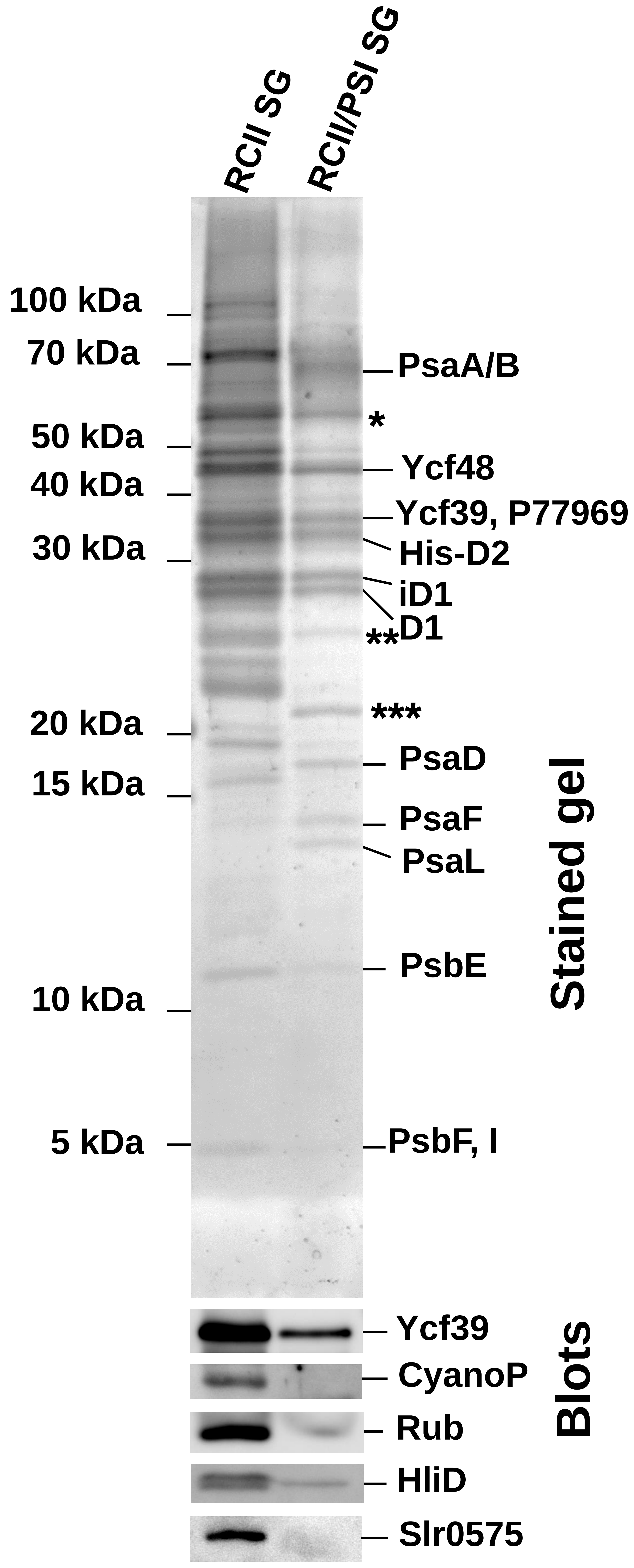

Supplementary Fig. 3 SDS-PAGE analysis of the upper sucrose gradient fraction containing RCII complexes (RCII SG) and the lower sucrose gradient fraction containing the RCII/PSI complex (RCII/PSI SG). Proteins identified by mass spectrometry and immunoblotting are indicated. 0.5 µg of Chl was loaded for each lane. Not all proteins detected by mass spectrometry are assigned to a stained band. * designates non-specific interacting elongation factor EF-Tu, ** and *** unknown proteins and P77969 is another unspecific contaminant delta-aminolevulinic dehydratase overlapping with the sharper band of Ycf39. This SDS-PAGE was repeated at least four times independently with the same results. Source data are provided at the end of this file.

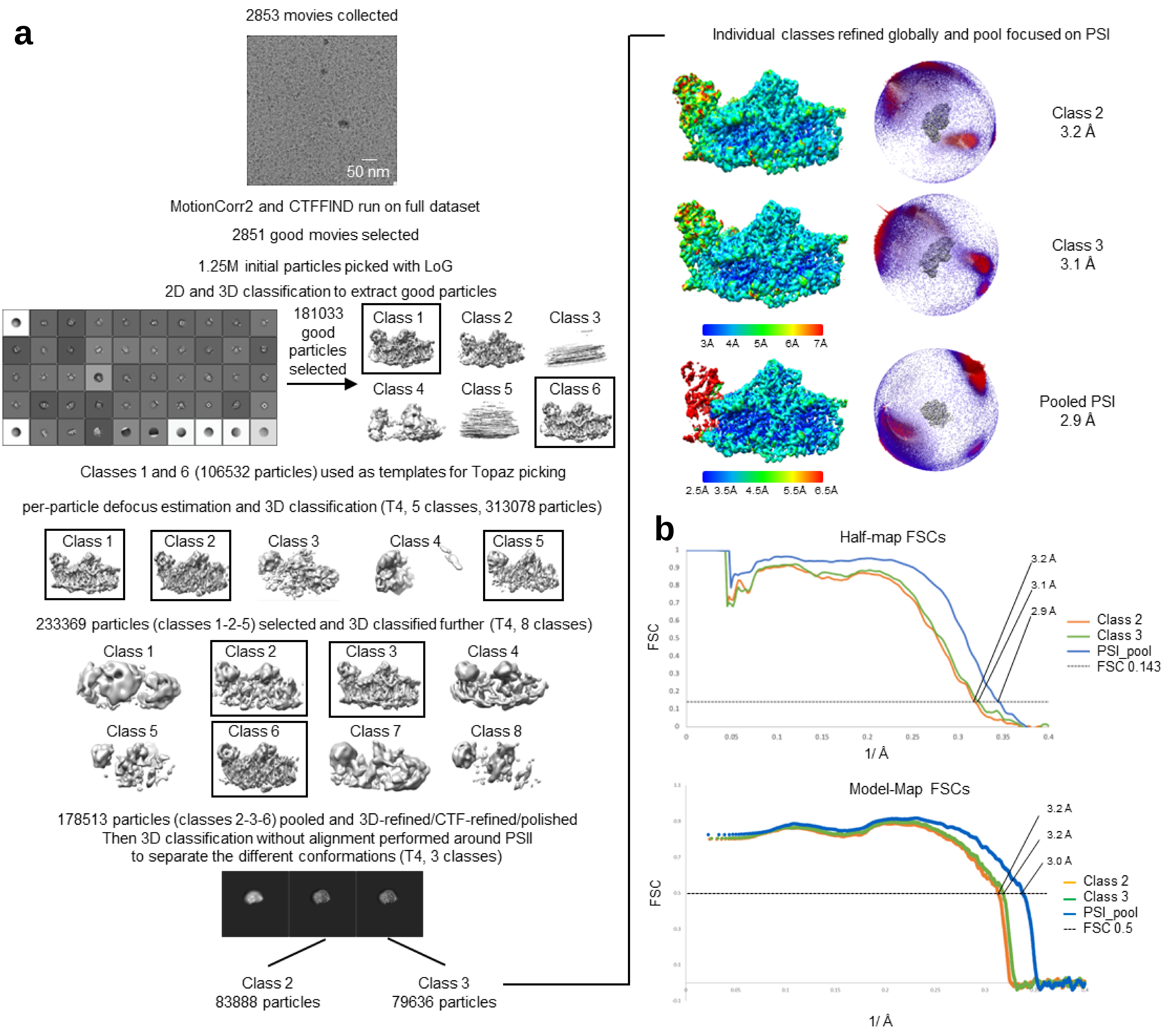

Supplementary Fig. 4 Processing pipeline, FSC curves and densities from the cryo-EM maps. a, Scheme of the processing pipeline showing a representative micrograph (out of the 2853 collected), the pre-processing and classification steps and the final maps, coloured by local resolution, with relative angular distribution plot and densities. **b,** Gold-standard half-map and model-map Fourier Shell Correlation (FSC) curves for the datasets.

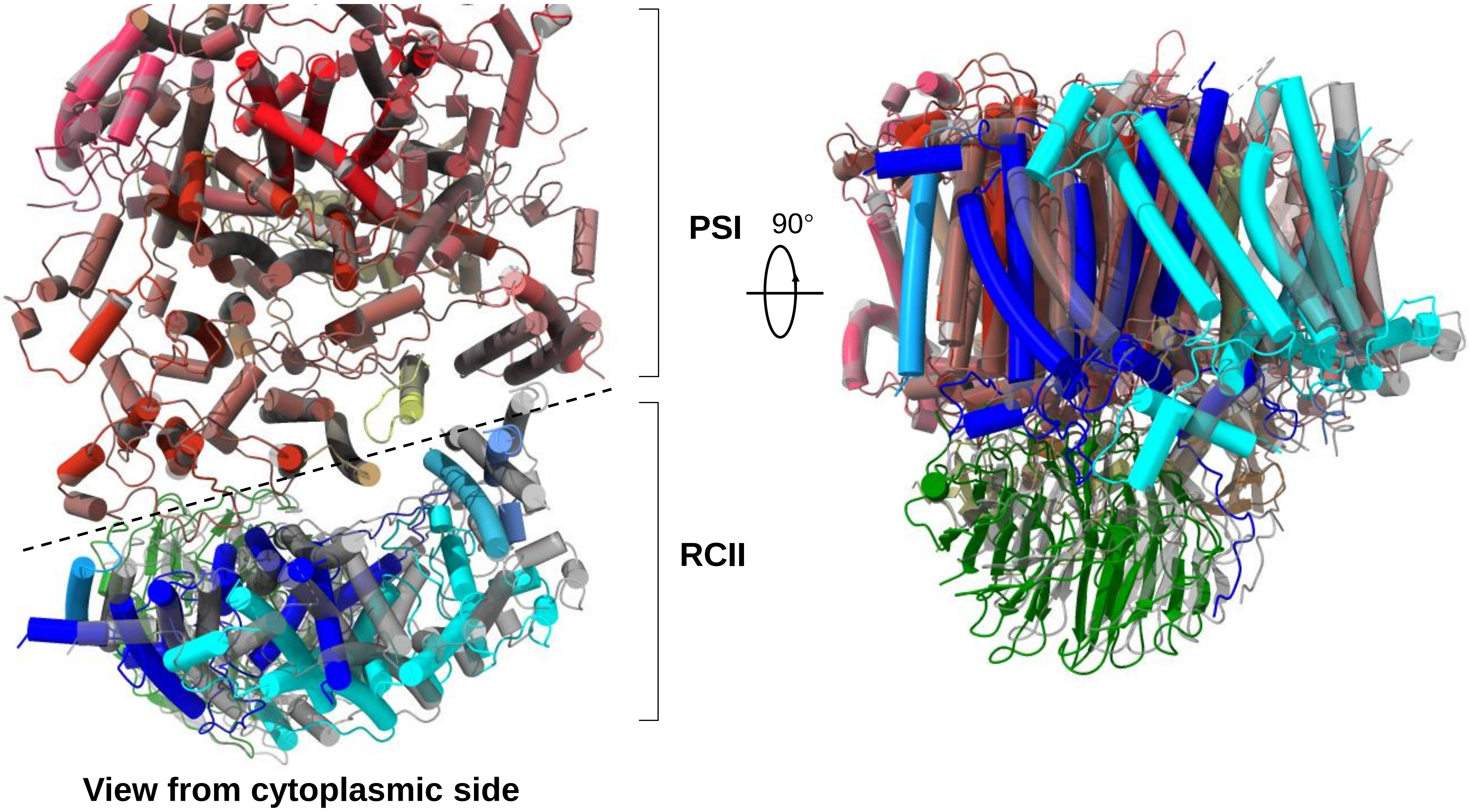

Supplementary Fig. 5 RCII position shifts in the lower resolution structure. The lower resolution structure (3.2 Å, shown as grey model) was superimposed to the higher resolution structure used for analysis in the manuscript (shown as coloured model) on PSI: the shift of RCII position, shown from the top view (cytoplasm) on the left and from the side view on the right, is visualized. The positions of D1 (blue), D2 (cyan) and Ycf48 (green) are shown.

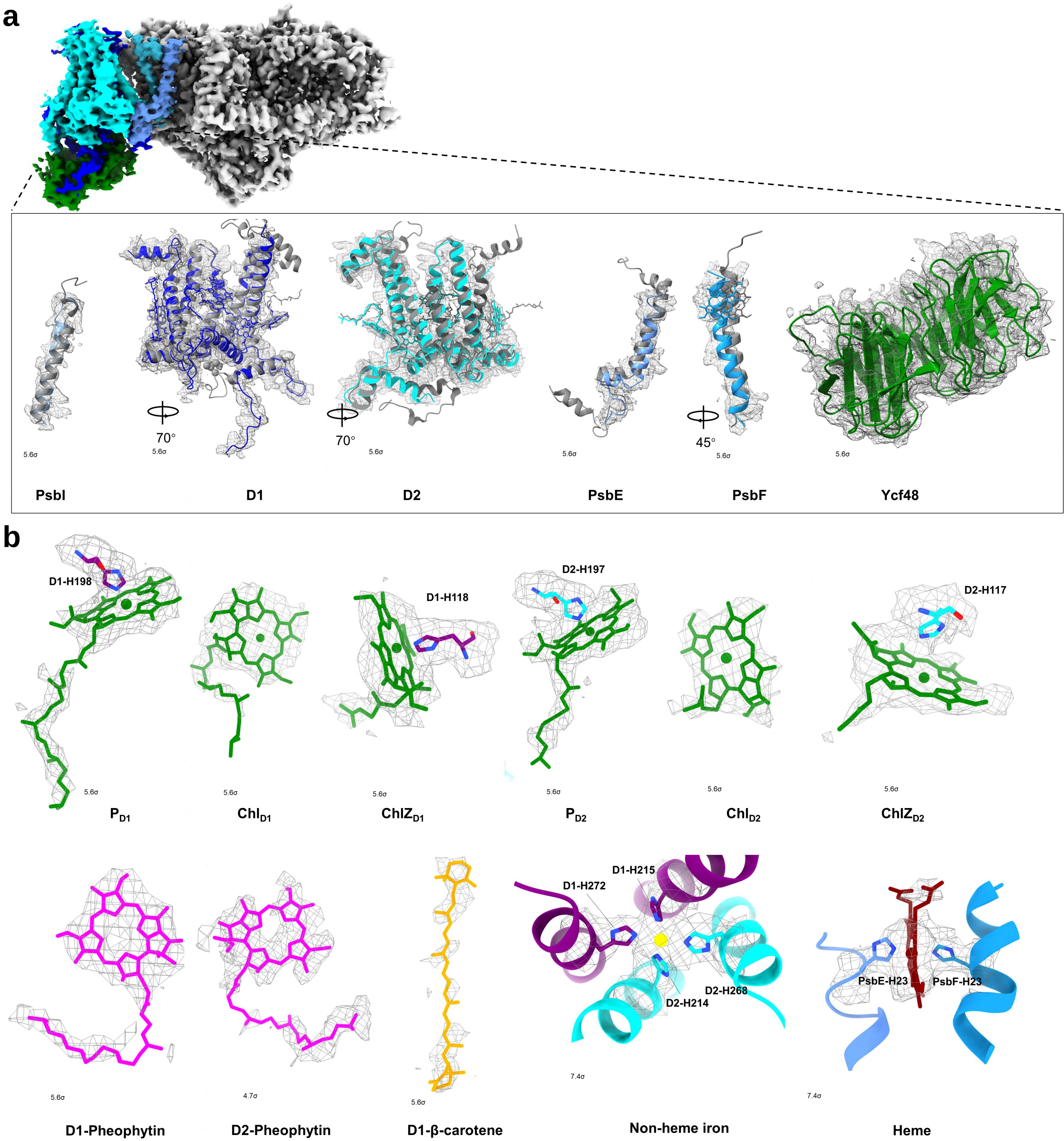

Supplementary Fig. 6 Class3 Cryo-EM densities and structural models of RCII subunits (a) and various cofactors (b). Modelled RCII subunits from the class 3 Ycf48/RCII/PSI structure (PDB ID: 8AM5) are in colour and in grey the equivalent subunits found in Syn6803 oxygen-evolving PSII (PDB ID: 7N8O. Representative density of subunits are contoured at 5.6σ. Chlorophylls, D1-pheophytin and D1-β-carotene are contoured at 5.6σ. D2-pheophytin is contoured at 4.7σ. Non-heme iron and heme are contoured at 7.4σ.

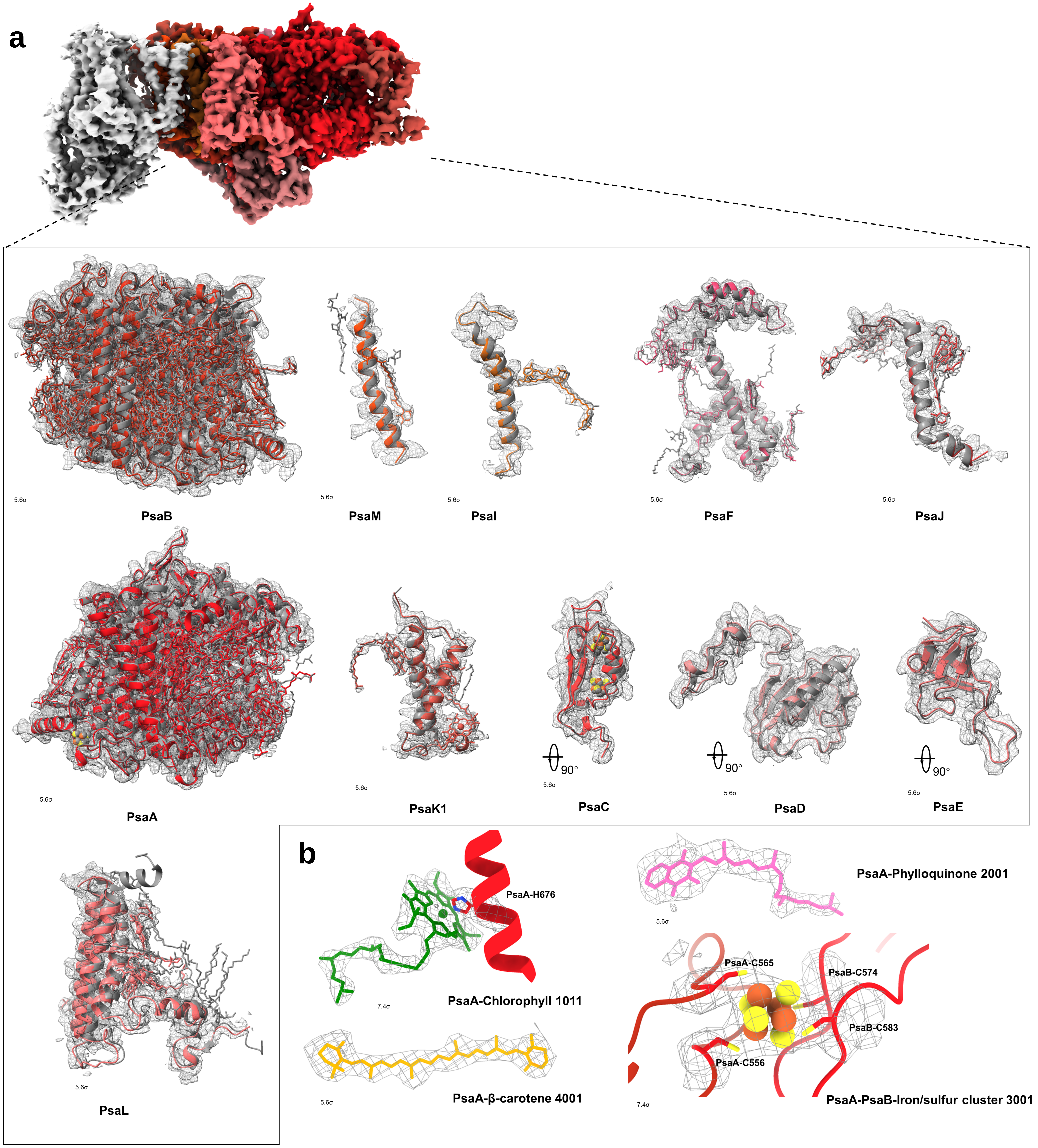

Supplementary Fig. 7 Class3 Cryo-EM densities and structural models of PSI subunits (a) and selected cofactors (b) in the PSI/RCII complex. Modelled subunits from the class 3 Ycf48/RCII/PSI structure (PDB ID: 8AM5) are shown in colour and in grey the equivalent found in the Syn6803 PSI monomer (PDB ID: 5OY0). Representative density of PSI subunits, PsaA-Chlorophyll 1011, PsaA-Phylloquinone 2001 are contoured at 5.6σ. PsaA-β-carotene 4001 and PsaA-PsaB-Iron/sulfur cluster 3001 are contoured at 7.4σ.

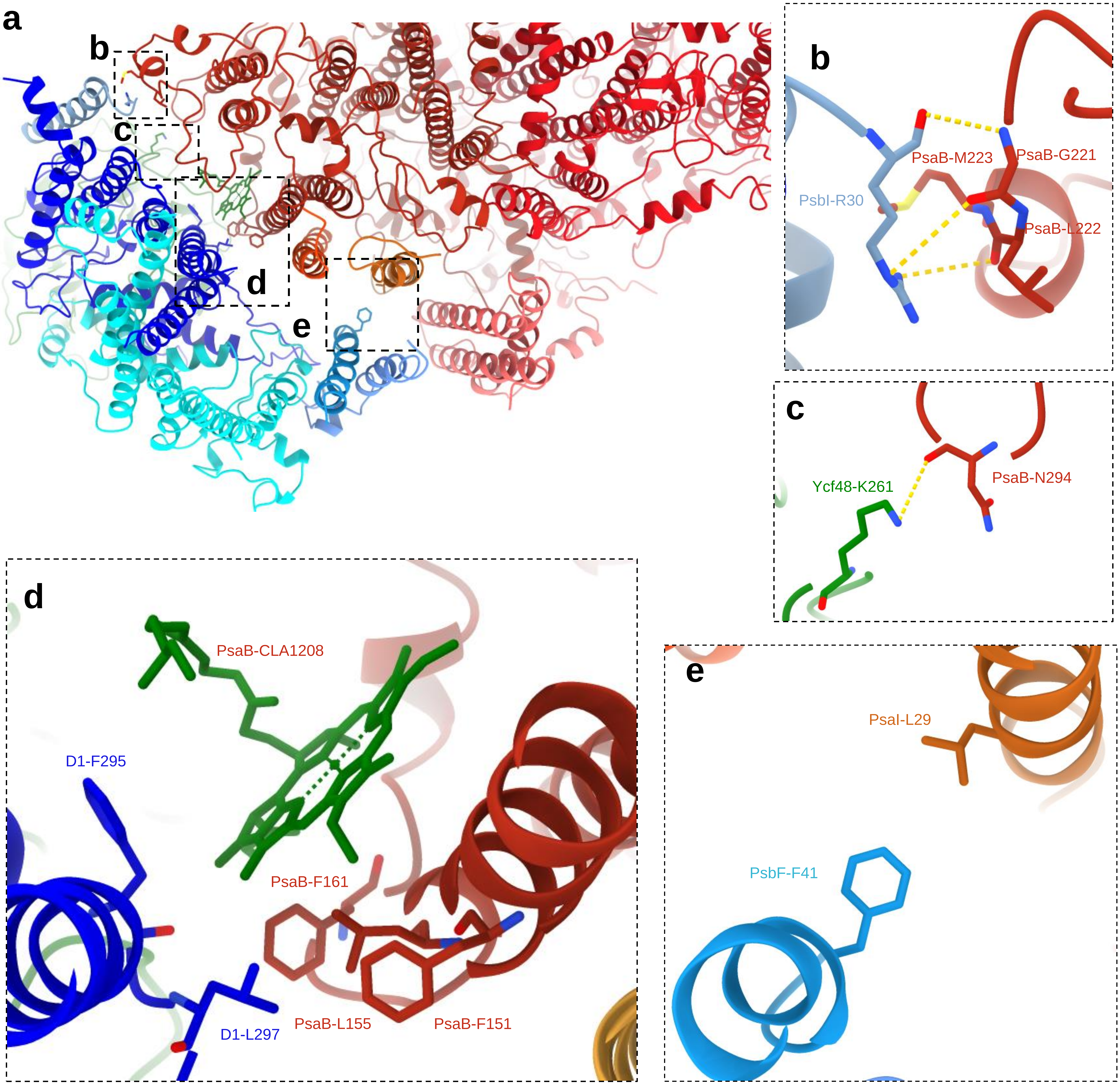

Supplementary Fig. 8 Interactions between RCII and PSI in the RCII/PSI complex. a, View of the RCII/PSI complex showing the regions at the interface of RCII and PSI under analysis. **b,** The PsbI-Arg30 backbone carbonyl oxygen, at the N-terminal end of PsbI, potentially H-bonds with the PsaB-Gly221 backbone nitrogen towards the end of the third helix of PsaB. **c,** Ycf48-Lys261, which is at the end of the fifth blade, may H-bond with PsaB-Asn294, a luminal residue at the end of the fourth PsaB helix. **d,** Possible hydrophobic interaction between D1 residues, D1-Phe295 and D1-Leu297, and PsaB residues, PsaB-Phe151, PsaB-Leu155, PsaB-Phe161 and chlorophyll 1208. **e,** C-terminal PsbF residue, PsbF-Phe41, may have hydrophobic interaction with PsaI-Leu29.

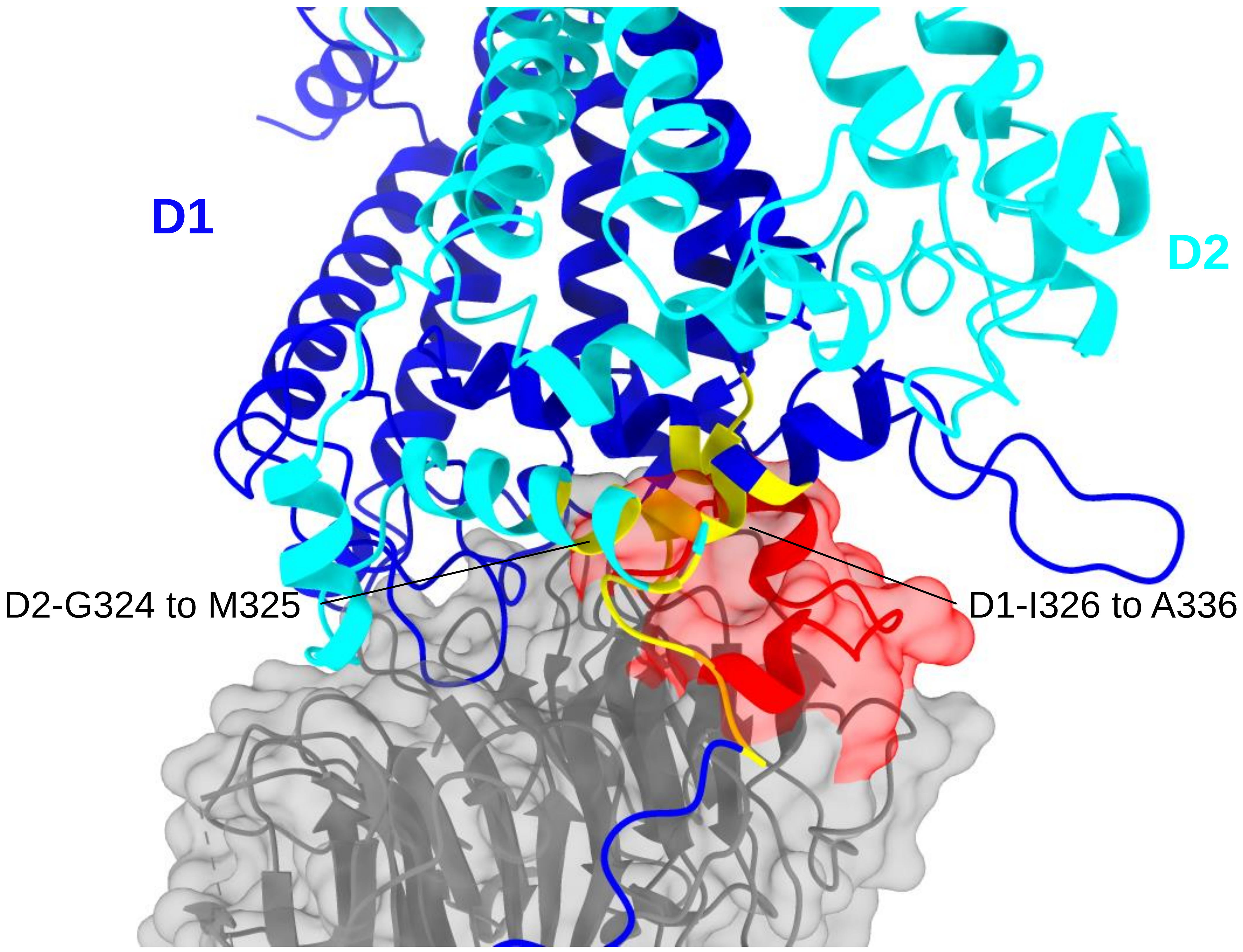

Supplementary Fig. 9 The Ycf48 eukaryotic loop clashes when modelled into the cyanobacterial RCII complex. Structure of *Cyanidioschyzon merolae* Ycf48 (PDB ID: 5OJ3, shown in grey) was superimposed onto Syn6803 Ycf48 in the PSI/RCII complex. The 19 amino-acid-residue insertion in the eukaryotic loop is coloured red. D1 is shown in blue and D2 in cyan. Sites of steric clash (D1-Ile326 to D1-Ala336 and D2-Gly324 to D2-Met325) are coloured yellow.

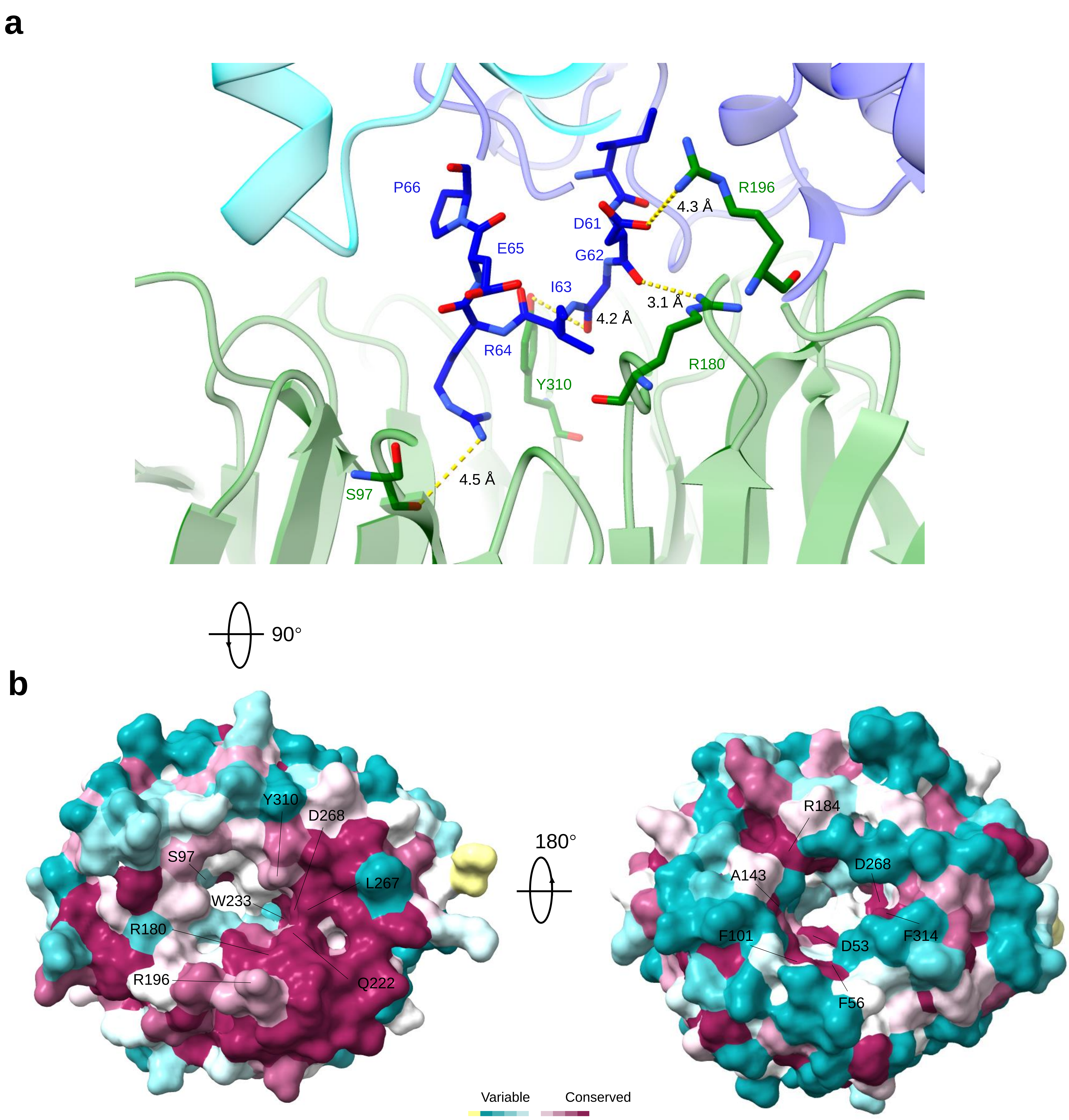

Supplementary Fig. 10 The Ycf48 channel. a, D1 loop at the exit of the Ycf48 channel. D1 in blue, Ycf48 in green and D2 in cyan. b, Identification of conserved residues within the Ycf48 channel viewed from the exit (left) and the entrance (right). Purple shows the most conserved regions and blue shows more variable regions. The first residue, Ycf48-His31, shown in yellow, is not conserved.

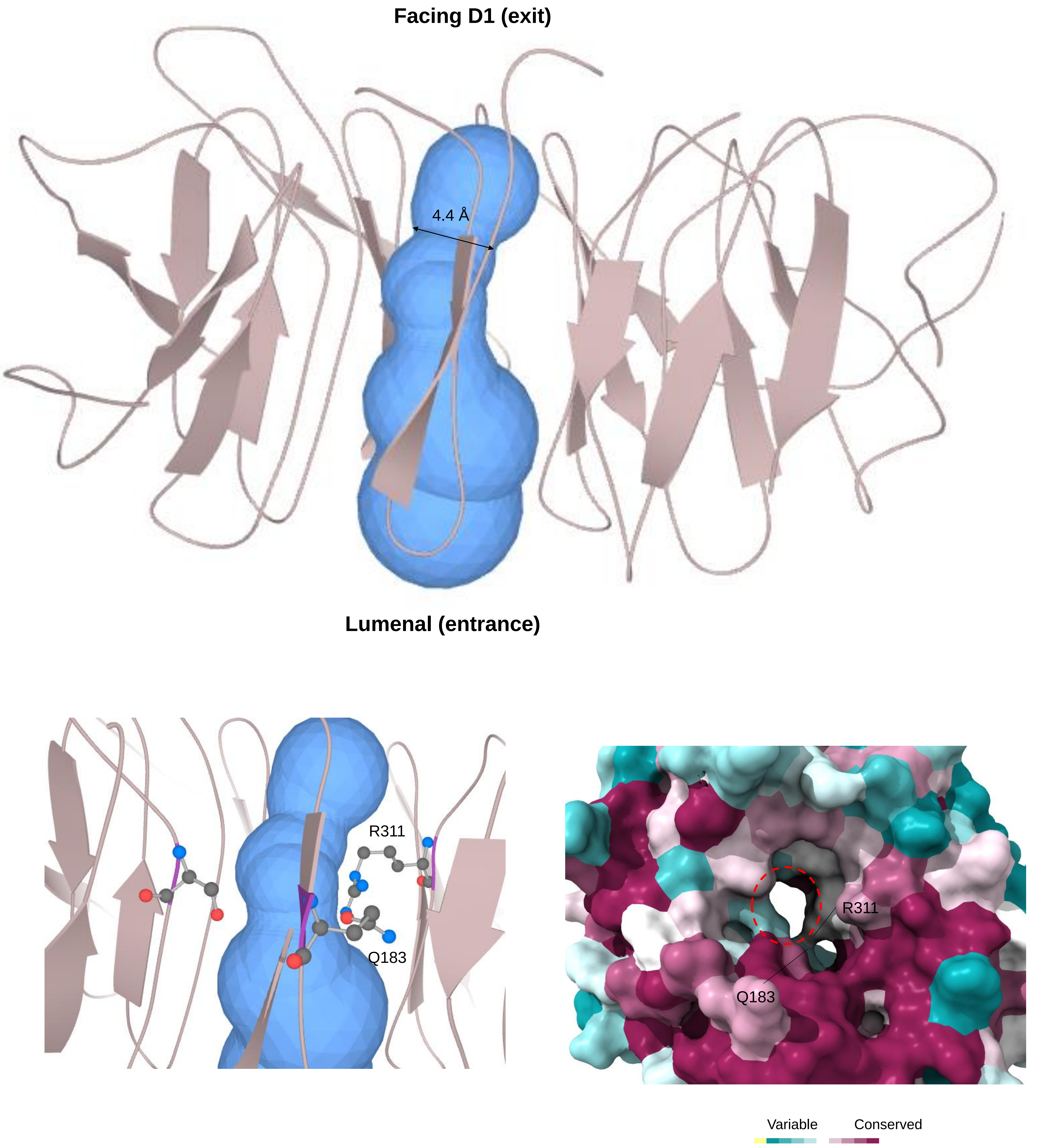

Supplementary Fig. 11 Ycf48 central channels. Ycf48 channels were analysed by MOLEonline (https://mole.upol.cz/). The central hole is split by R311 and Q183 into two tunnels, the bottleneck diameter of the widest tunnel is shown. Lumenal side of the channel is defined as the ‘entrance’ and the side facing D1 is the ‘exit’. Minor tunnels not shown.

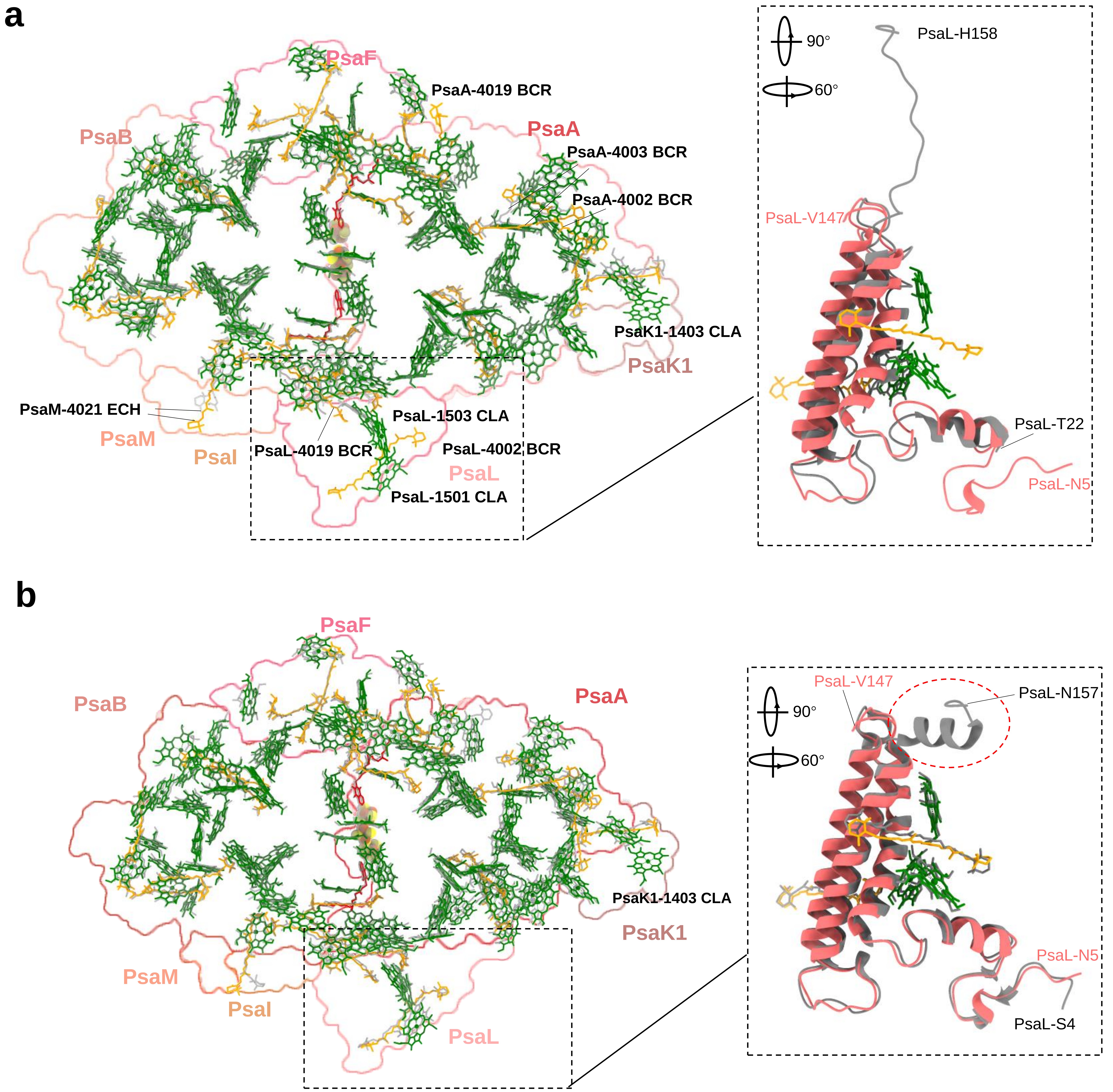

Supplementary Fig. 12 Comparison of pigments PSI in the RCII/PSI complex with published structures of monomeric and trimeric PSI from Syn6803. a, Top view comparison of PSI cofactors in the PSI/RCII complex and the Syn6803 PSI monomer (PDB ID: 6HQB). Chlorophylls are shown in green, carotenoids in yellow and phylloquinones in red. Three additional chlorophylls (CLA) and four additional β-carotenes (BCR) are indicated in bold. One PsaM echinenone (PsaM-4021 ECH) and one PsaA β-carotene (PsaA-4003 BCR) show different positions. The dashed box shows differences between PsaL in the PSI/RCII complex (pink) and PSI monomer (grey). **b,** Top view comparison of PSI cofactors in the RCII/PSI complex and the monomer found in the Syn6803 PSI trimer (PDB ID: 5OY0). Dashed box compares the structures of PsaL in the RCII/PSI complex (coloured pink) and in the trimer (in grey). The short C-terminal helix of PsaL, which is involved in PSI trimerization, cannot be modelled in the PSI/RCII structure. Additional chlorophyll indicated in bold.

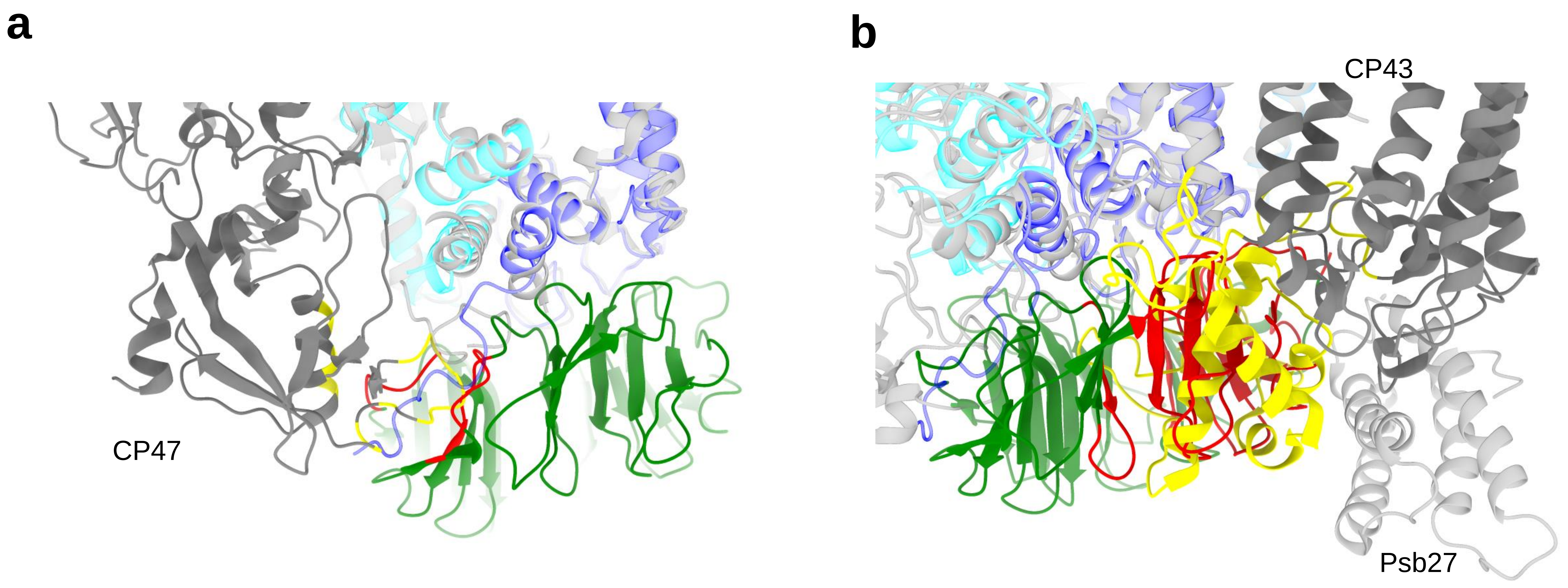

Supplementary Fig. 13 Potential steric clashes between CP47 and CP43 when the structure of Ycf48 is inserted into the structures of RC47 and the Psb27/PSII complex. a, Loop region of CP47 (CP47-Pro382 to CP47-Lys389, in yellow) and several luminal helix residues (CP47-Ser391, Glu393, Gln394, Pro415, Lys418 and Arg422, in yellow) clash with blade 3 residues of Ycf48 (Ser131-Ala137, Asp154-Tyr159, Ala170, Glu173-Ala175, in red) when *T. vulcanus* RC47 (PDB ID:7DXA) is superimposed on the structure of RCII. Ycf48 is shown in green. D1 and D2 in the RCII/PSI complex are shown in transparent blue and cyan, respectively. RC47 subunits are shown in grey, CP47 is shown in solid and other subunits are transparent. **b,** *T. vulcanus* Psb27/PSII (PDB ID:7CZL) was superimposed onto RCII. Psb27/PSII subunits are shown in grey, CP43 is solid and other subunits are transparent. Ycf48 is shown in green. D1 and D2 in RCII/PSI complex are shown in transparent blue and cyan, respectively. The large lumenal region of CP43 (Pro217-Asn228, Pro304-Val420, in yellow) clashes with blades 4, 5 and 6 of Ycf48 (His31, Arg180-Arg189,Tyr200, Asn214-Asp299, Tyr310-Phe314, in red).

	Subunit name and Uniprot code	Size (Da) Length (AA)	MS Intensity	Blot	Chain name in the model	Range built/total residues	Unmodeled residues	% modelled residues	Cofactors	Notes
RCII	**D1, PsbA P16033**	39695 360	90237000	+ +	A	13-224,267-344/1-344	1-12, 225-266	84.3	chlorophyll, non-heme iron, β-carotene, pheophytin	Cytoplasmic loop around non-heme iron unmodeled
	**D2, PsbD P09192**	39466 352	112990000	++	D	32--222,244-330/1-352	1-31, 223-243,331-352	79.0	chlorophyll, non-heme iron, pheophytin	Cytoplasmic loop around non-heme iron unmodeled
	**PsbE P09190**	9442 81	28092000	++	E	20-56/2-81	2-19, 57-81	46.3	heme	N-terminal and C-terminal short helices unmodeled
	**PsbF P09191**	4929 44	26176000	++	F	17-44/2-44	2-16	65.1	heme	N-terminal tail unmodeled
	**PsbI Q54697**	4306 38	n.d.	++	I	2-30/1-38	1, 31-38	76.3		C-terminal tail unmodeled
	**Ycf48 P73069**	37267 342	279630000	++	S	31-333/29-339	29-30, 334-339	97.1		Ycf48 is lipidated at Cys29, three C-terminal residues are removed
	**RubA P73068**	12570 115	31088000	+						
	**Ycf39 P74429**	36496 326	11390000	+						
	**HliD P73563**	6472 57	630300	+						
	**RCII**					965/1168		82.6		
PSI	**PsaA P29254**	82950 751	26052000	++	a	11-751/1-751	1-10	98.7	chlorophyll, phylloquinone, β-carotene, Fe_4S_4 cluster	
	**PsaB P29255**	81292 731	8619200	++	b	3-731/2-731	2	99.9	chlorophyll, phylloquinone, β-carotene, Fe_4S_4 cluster, canthaxanthin	
	**PsaC P32422**	8828 81	9414800	++	c	2-81/2-81		100.0	Fe_4S_4 cluster	
	**PsaD P19569**	15644 141	55535000	++	d	3-141/2-141	2	99.3		
	**PsaE P12975**	8145 74	25510000	n.a.	e	2-70/2-74	71-74	94.5		
	**PsaF P29256**	18249 165	58829000	++	f	24-165/24-165		100.0	chlorophyll, zeaxanthin	1-23 is signal peptide
	**PsaI Q55330**	4413 40		n.a.	i	1-40/1-40		100.0	β-carotene, 3 'OH echinenone	
	**PsaJ Q55329**	4532 40		n.a.	j	1-40/1-40		100.0	chlorophyll, β-carotene, zeaxanthin	
	**PsaK1 P72712**	8644 86	335670	n.a.	k	9-83/1-86	1-8, 84-86	87.2	chlorophyll, β-carotene	
	**PsaK2 P74564**	9306 90	2946400	n.a.						
	**PsaL P37277**	16624 157	27715000	++	l	5-147/1-157	1-4, 148-157	91.1	chlorophyll, β-carotene	C-terminal helix unmodeled
	**PsaM P72986**	3380 31	3305200	n.a.	m	1-31/1-31		100.0	echinenone	
	**PSI**					2229/2270		98.2		
**Total**	RCII/PSI					3194/3438		93.0		

Supplementary Table 1 List of PSII and PSI subunits detected in the RCII/PSI complex by mass spectrometry (MS) and immunoblotting (Blot) and subunit composition of the deposited model. The MS analysis of proteins precipitated from the preparation was performed using NanoElute UHPLC (Bruker) on-line coupled to a high-resolution mass spectrometer (Bruker Impact HD). n.d. means not detected, n.a. means not analysed. ++ designates abundant most probably stoichiometric components, + less abundant components.

.

	#1 class2 (EMDB-15618) (PDB 8ASL)	#1 class3 (EMDB-15522) (PDB 8AM5)	#3 PSI_focus_ref (EMDB-15621) (PDB 8ASP)
**Microscope**	Glacios	Glacios	Glacios
**Camera**	Falcon 3 linear	Falcon 3 linear	Falcon 3 linear
**Magnification**	120000X	120000X	120000X
**Voltage (kV)**	200	200	200
**Electron exposure (e–/Å^2)**	91	91	91
**Automation software**	EPU	EPU	EPU
**Number of frames**	58	58	58
**Defocus range (μm)**	c.a. -1.2 to -2.5	c.a. -1.2 to -2.5	c.a. -1.2 to -2.5
**Pixel size (Å)**	1.22	1.22	1.22
**Symmetry imposed**	C1	C1	C1
**Number of micrographs**	2853	2853	2853
**Initial particle images (no.) from Topaz**	313078	313078	313078
**Final particle images (no.)**	83888	79636	163524
**Map resolution (Å) at 0.143 FSC threshold**	3.2	3.1	2.9
**Local resolution range (Å)**	2.7-9.5	2.7-9.2	2.7-5.0
**Refinement**			
**Initial model used (PDB code)**	RCII (6WJ6); PSI (5OY0); Ycf48 (2XBG)	RCII (6WJ6); PSI (5OY0); Ycf48 (2XBG)	(5OY0)
**Refinement package**	Phenix, real space	Phenix, real space	Phenix, real space
**Model resolution (Å) at 0.5 FSC threshold**	3.2	3.2	3.0
**Cross-correlation**			
**Mask**	0.86	0.87	0.89
**Volume**	0.85	0.86	0.88
**Map sharpening B factor (Å^2)**	-60.2	-55.3	-54.6
**Model composition**			
**Non-hydrogen atoms**	32740	32747	24597
**Protein residues**	3198	3194	2230
**Ligands**	145	146	134
**B factors (Å^2) (mean)**			
**Protein**	89.15	93.00	69.14
**Ligand**	66.23	69.11	65.03
**R.m.s deviations**			
**Bond lengths (Å)**	0.003	0.003	0.002
**Bond angles (°)**	0.583	0.573	0.597
**Validation**			
**MolProbity score**	1.86	1.82	1.87
**EMRinger score**	2.66	2.62	2.58
**Clashscore**	14.79	13.81	13.55
**Poor rotamer (%)**	0.08	0.08	1.34
**C-beta outliers %**	0	0	0
**CaBLAM outliers (%)**	1.54	1.54	1.51
**Ramachandran plot**			
**Favored (%)**	96.93	96.99	97.28
**Allowed**	3.07	3.01	2.72
**Disallowed (%)**	0	0	0

Supplementary Table 2 Cryo-EM data collection, refinement and validation statistics

ConSurf Color-Coded MSA		
001 Input_pdb_ATOM_S	051 UniRef90_UPI001CEC8D81_29_329	101 UniRef90_A0A651ENY8_32_331
002 UniRef90_A0A2W7CY78_30_329	052 UniRef90_A0A6N8FTV1_28_326	102 UniRef90_UPI0018F04C05_40_341
003 UniRef90_E0UC10_29_327	053 UniRef90_UPI000AC320BB_26_326	103 UniRef90_A0A0M1JRQ4_27_338
004 UniRef90_A0A401IC87_23_323	054 UniRef90_UPI001ABA873B_27_327	104 UniRef90_K9YCU5_27_328
005 UniRef90_B7K627_29_329	055 UniRef90_K1WAK8_28_329	105 UniRef90_A0A0P7ZT17_27_328
006 UniRef90_A8YAZ5_23_324	056 UniRef90_K9QUS4_26_326	106 UniRef90_A0A1L9QX77_29_332
007 UniRef90_K9Q064_35_335	057 UniRef90_UPI001881EDF7_27_327	107 UniRef90_A0A251W9U3_41_341
008 UniRef90_A0A6P2AGS2_23_323	058 UniRef90_A0A2I8A2S4_26_326	108 UniRef90_UPI0008F9BB1D_27_328
009 UniRef90_A0A0D6ASH1_27_328	059 UniRef90_A0A7C3KBR3_29_328	109 UniRef90_K9UWE4_27_330
010 UniRef90_K9XZ78_30_327	060 UniRef90_A0A6P0R9T9_29_329	110 UniRef90_UPI0014774849_27_328
011 UniRef90_A0A1Z4RRI4_27_327	061 UniRef90_A0A2T1DYF7_33_332	111 UniRef90_A0A2W7ADG5_35_330
012 UniRef90_UPI00034D22B6_43_343	062 UniRef90_A0A2T2RPB8_34_330	112 UniRef90_A0A352JCS3_36_330
013 UniRef90_L8LPA3_27_323	063 UniRef90_D7E3B7_27_328	113 UniRef90_A0A7Y5I850_40_334
014 UniRef90_A0A1E5QTE7_33_331	064 UniRef90_A0A7C3ZFV4_24_325	114 UniRef90_K9SHI7_74_369
015 UniRef90_UPI0005674AC8_28_327	065 UniRef90_A0A846BN71_27_328	115 UniRef90_A0A0M2PW93_19_326
016 UniRef90_UPI000B59F958_29_330	066 UniRef90_A0A6G3Z5J0_32_329	116 UniRef90_L8MW52_37_331
017 UniRef90_A0A3M1PGN5_29_332	067 UniRef90_A0A1Z4IEM3_26_326	117 UniRef90_A0A1J0AGC8_23_324
018 UniRef90_A0A3C0N8Z6_29_327	068 UniRef90_A0A6I5NL56_29_332	118 UniRef90_A0A2N5JQV3_26_319
019 UniRef90_A0A3M9Z559_29_329	069 UniRef90_A0A856MKL5_28_328	119 UniRef90_A0A6P2ANJ9_27_273
020 UniRef90_A0A352AIT5_27_327	070 UniRef90_A0A846FDN3_28_328	120 UniRef90_K9ST67_39_334
021 UniRef90_UPI00177FE0CF_29_327	071 UniRef90_A0A654SFQ5_26_326	121 UniRef90_A0A2W1JSZ6_19_316
022 UniRef90_A0A1E5QLC2_27_328	072 UniRef90_A0A1Z4K130_27_328	122 UniRef90_A0A3D1PJW6_1_219
023 UniRef90_A0A351KYP9_27_328	073 UniRef90_K9VI09_17_315	123 UniRef90_A0A3G1IWH9_105_402
024 UniRef90_UPI001682A563_30_329	074 UniRef90_A0A1Z4QEU3_27_327	124 UniRef90_A0A4V1DHX1_30_329
025 UniRef90_UPI0016868E07_34_330	075 UniRef90_A0A6I5PBD7_37_343	125 UniRef90_A0A8E6CBC1_45_341
026 UniRef90_UPI0013918DD4_28_328	076 UniRef90_Q8YQI3_26_325	126 UniRef90_A0A7T1MLW0_39_338
027 UniRef90_UPI001CA6F719_28_327	077 UniRef90_UPI00168100E0_27_328	127 UniRef90_A0A2H4ZPR6_37_331
028 UniRef90_UPI0017467DBC_29_328	078 UniRef90_K9ZE53_27_328	128 UniRef90_A0A4P5VDW6_43_342
029 UniRef90_UPI00168676E7_29_330	079 UniRef90_A0A2A2TKU5_27_328	129 UniRef90_A0A7T1ME64_36_335
030 UniRef90_UPI001685FCEF_28_330	080 UniRef90_A0A1Z4H6N4_27_328	130 UniRef90_Q05QX8_34_332
031 UniRef90_UPI001689F6D9_26_326	081 UniRef90_A0A6H2NNI6_30_329	131 UniRef90_A0A6N2ESM7_34_329
032 UniRef90_A0A7Z9C3M1_26_327	082 UniRef90_A0A1U7J9G4_31_329	132 UniRef90_A3YTN1_30_329
033 UniRef90_A0A1J1LH67_26_327	083 UniRef90_A0A1Z4KXU9_27_327	133 UniRef90_UPI000E0E107F_35_328
034 UniRef90_A0A1Q8Z802_29_328	084 UniRef90_A0A1Z4UYB9_27_328	134 UniRef90_A0A081GIU4_40_345
035 UniRef90_UPI001682F8DA_29_330	085 UniRef90_A0A0T7BVJ1_31_328	135 UniRef90_K9P961_39_344
036 UniRef90_UPI00145F2C07_27_328	086 UniRef90_A0A2Z6C9H0_26_327	136 UniRef90_A0A2P5FM67_91_411
037 UniRef90_UPI001685B803_27_327	087 UniRef90_A0A2W4WA90_31_329	137 UniRef90_A0A200QNT3_106_424
038 UniRef90_A0A3D1PEW8_27_327	088 UniRef90_A0A2T1C9S7_27_325	138 UniRef90_UPI0018E35B31_34_333
039 UniRef90_K9XEF4_27_326	089 UniRef90_K9S4G6_28_331	139 UniRef90_A0A7G7KMD8_26_325
040 UniRef90_A0A1C0VTD6_30_330	090 UniRef90_UPI001C0287F7_37_343	140 UniRef90_A0A2W6Z147_33_329
041 UniRef90_UPI001683BDE8_27_327	091 UniRef90_A0A0C1UQC2_29_329	141 UniRef90_A0A176WI58_117_434
042 UniRef90_A0A2T1DP99_24_324	092 UniRef90_A0A1Z4JNW8_43_343	142 UniRef90_A0A560L2Y9_35_329
043 UniRef90_A0YVX5_26_327	093 UniRef90_UPI00034BB812_27_327	143 UniRef90_A0A2D8TV09_29_332
044 UniRef90_F4XMG1_25_323	094 UniRef90_A0A2W4XCM6_50_347	144 UniRef90_A0A6J1KSF8_83_401
045 UniRef90_UPI0019528272_26_336	095 UniRef90_A0A0P7ZMK7_41_338	145 UniRef90_A0A7G8ER83_45_340
046 UniRef90_UPI001682BF3F_29_330	096 UniRef90_B8HVD6_30_326	146 UniRef90_A0A3S3P098_77_397
047 UniRef90_UPI00188159A5_27_328	097 UniRef90_A0A3M1T1R3_27_328	147 UniRef90_A0A3R7NV24_28_328
048 UniRef90_UPI00168271E9_27_328	098 UniRef90_K9PGS4_27_327	148 UniRef90_A0A2G4HIL4_36_335
049 UniRef90_A0A2P8W4M1_31_329	099 UniRef90_A0A168WI24_27_327	149 UniRef90_A0A5J9TAB8_67_385
050 UniRef90_A0A098TL13_28_328	100 UniRef90_UPI000EFB0FEA_38_338	150 UniRef90_Q3B0C5_29_328

Supplementary Table 3. 150 sequences of Ycf48 homologs used for multiple sequence alignment (MSA) in ConSurf.

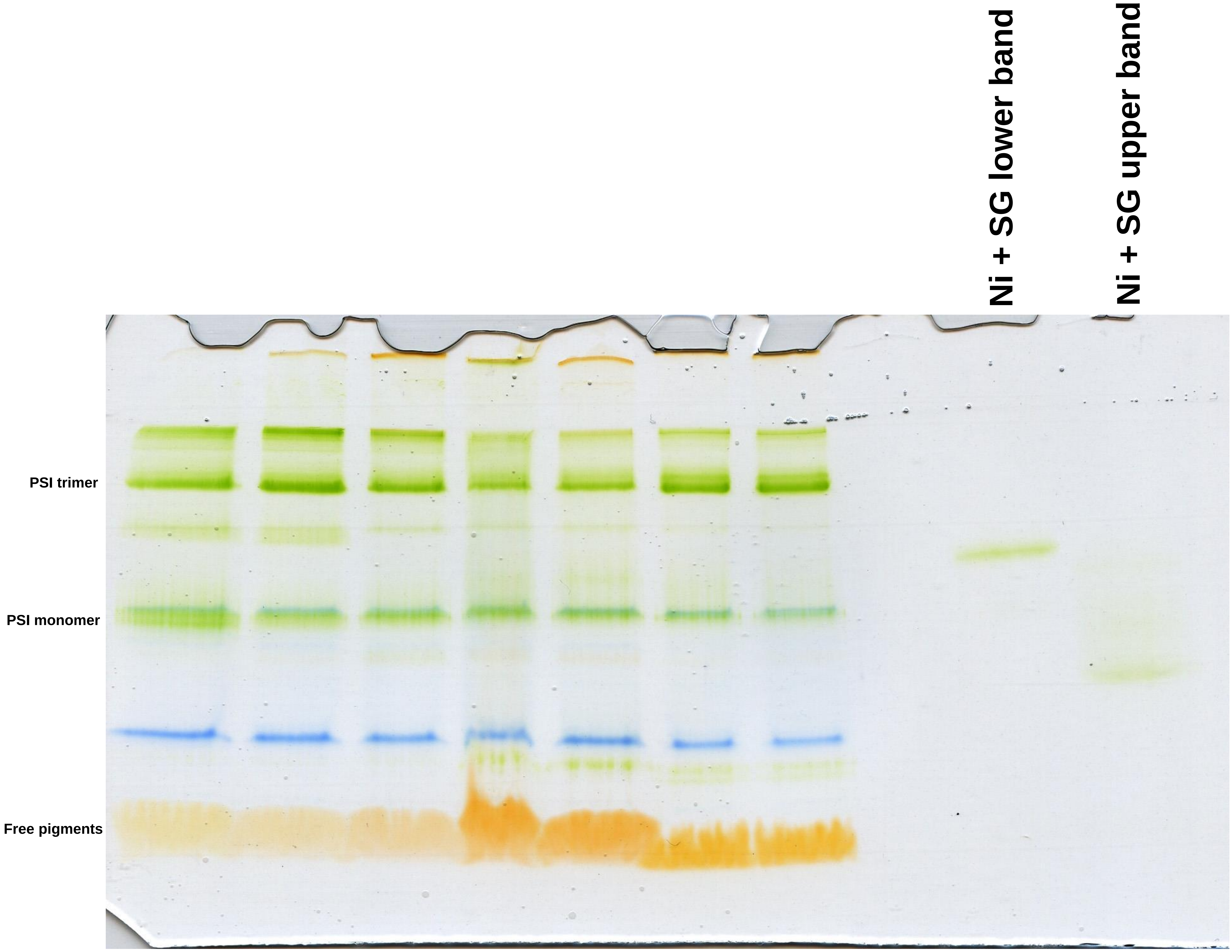

Uncropped data for Supplementary Fig. 2.

Clear-native gel analysis of the purified fractions from the sucrose gradient. The upper band (Ni +SG upper band) and lower band (Ni +SG lower band) are designated. Other lanes are of solubilized thylakoid membranes.

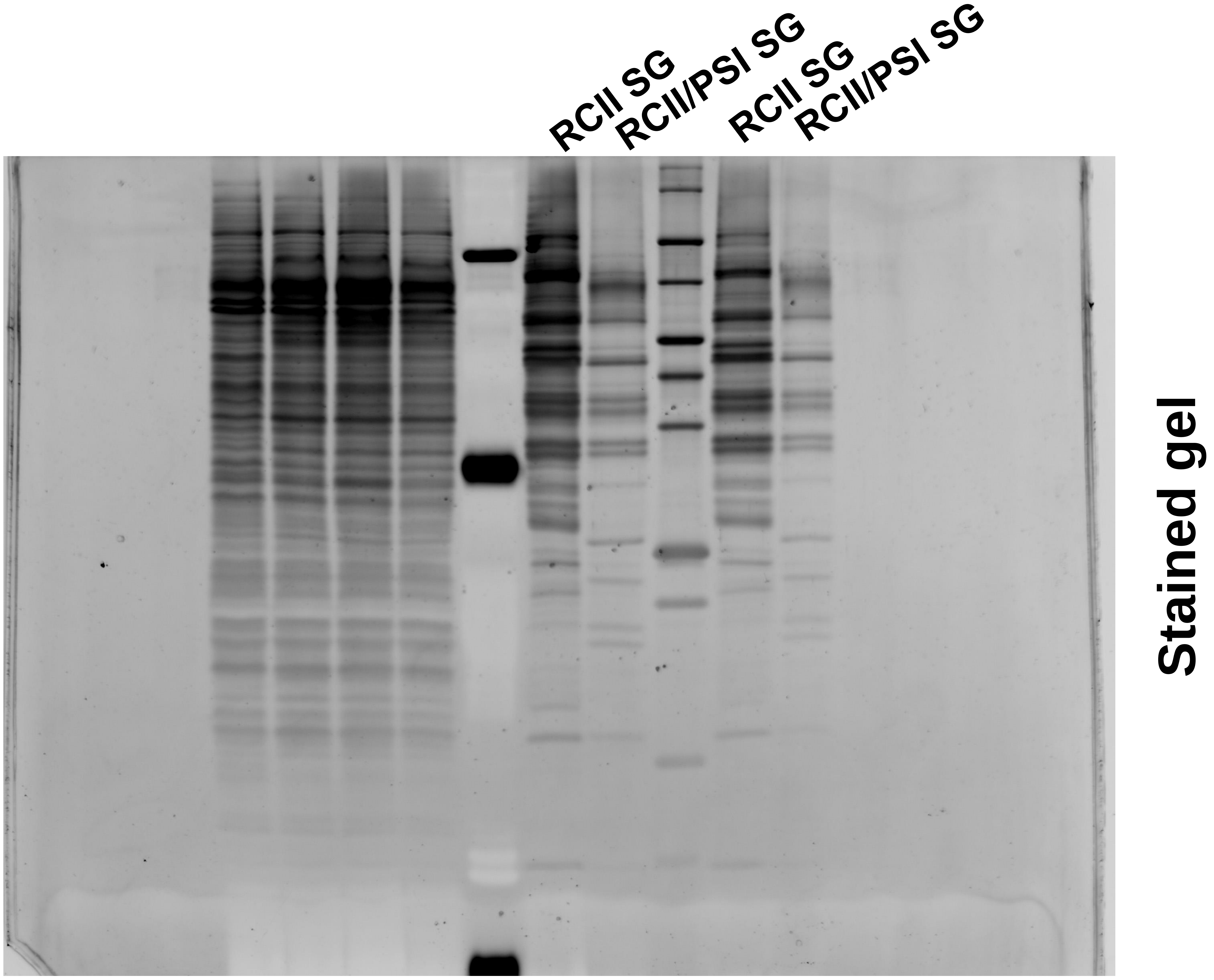

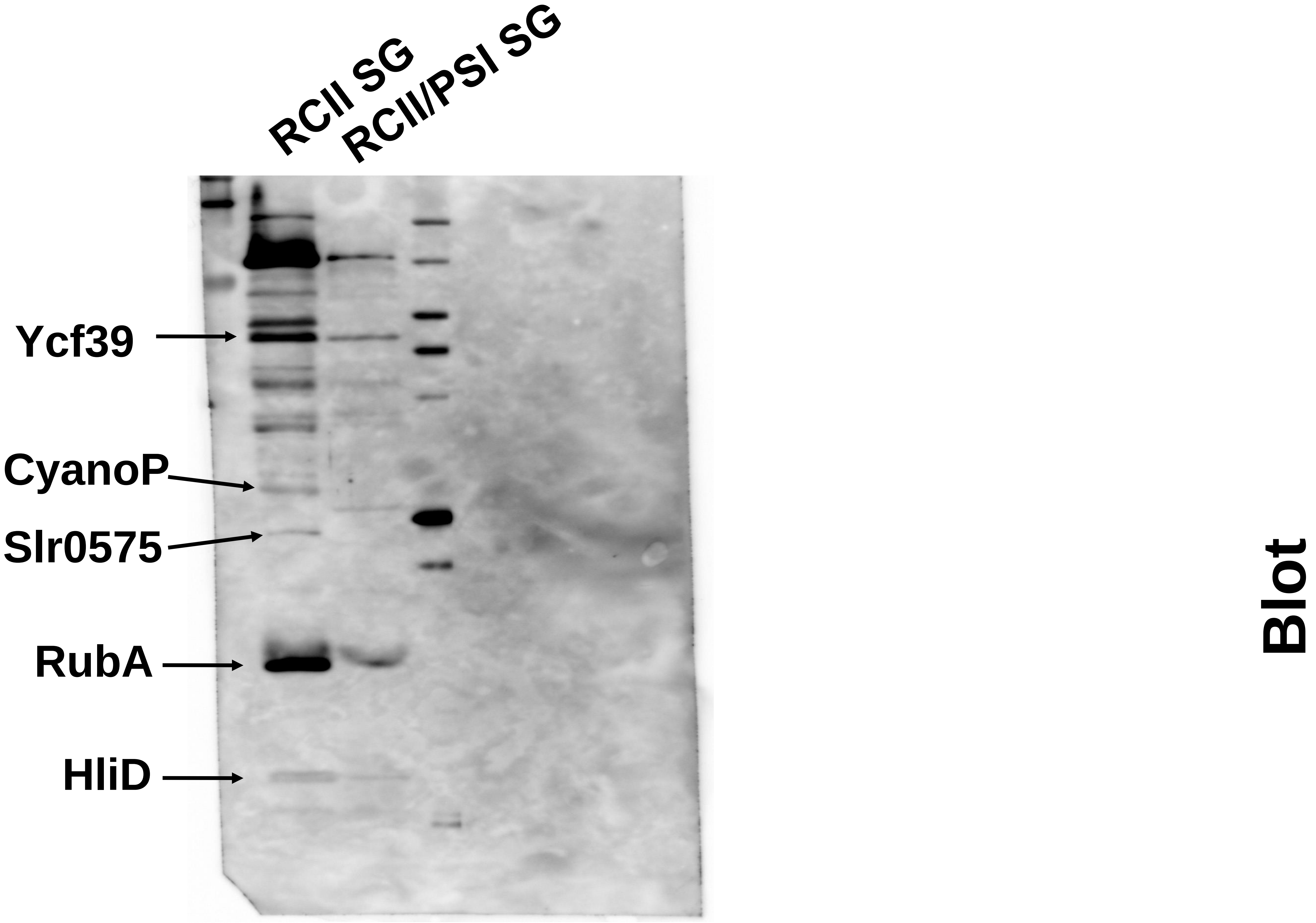

Uncropped data for Supplementary Fig. 3. Stained SDS-PAGE gel (Stained gel) and immunoblot (blot) of gel portion with the upper sucrose gradient fraction containing RCII complexes (RCII SG) and the lower sucrose gradient fraction containing the RCII/PSI complex (RCII/PSI SG). The immunoblot (blot) was probed sequentially with antibodies specific for Slr0575, then RubA, then HliD, then Ycf39 and finally CyanoP. The final image is shown.
